# Supplementary material for: Determinants shaping the nanoscale architecture of the mouse rod outer segment
Source: eLife. 2021 Dec 21;10:e72817. doi: 10.7554/eLife.72817 (PMC8758146; doi:10.7554/eLife.72817)
Supplement: Transparent reporting form [file elife-72817-transrepform1.pdf]

eLIFE
1st Floor
24 Hills Road
Cambridge CB2 1JP, UK
P 01223 855340
W elifesciences.org
T @elife

# *eLife's* transparent reporting form

We encourage authors to provide detailed information *within their submission* to facilitate the interpretation and replication of experiments. Authors can upload supporting documentation to indicate the use of appropriate reporting guidelines for health-related research (see EQUATOR Network), life science research (see the BioSharing Information Resource), or the ARRIVE guidelines for reporting work involving animal research. Where applicable, authors should refer to any relevant reporting standards documents in this form.

If you have any questions, please consult our Journal Policies and/or contact us: editorial@elifesciences.org.

**Sample-size estimation**

- You should state whether an appropriate sample size was computed when the study was being designed
- You should state the statistical method of sample size computation and any required assumptions
- If no explicit power analysis was used, you should describe how you decided what sample (replicate) size (number) to use

Please outline where this information can be found within the submission (e.g., sections or figure legends), or explain why this information doesn't apply to your submission:

No explicit power analysis was used. The sample size was limited by the technical constraints of high-quality cryo-ET imaging. The number of used tomograms and extracted subvolumes for averaging of ROS disk rims are listed Table 1. 7000 Connectors were segmented in 5 Volta phase plate tomograms (Table 1).

**Replicates**

- You should report how often each experiment was performed
- You should include a definition of biological versus technical replication
- The data obtained should be provided and sufficient information should be provided to indicate the number of independent biological and/or technical replicates
- If you encountered any outliers, you should describe how these were handled
- Criteria for exclusion/inclusion of data should be clearly stated
- High-throughput sequence data should be uploaded before submission, with a private link for reviewers provided (these are available from both GEO and ArrayExpress)

Please outline where this information can be found within the submission (e.g., sections or figure legends), or explain why this information doesn't apply to your submission:

**Methods: Cryo-transmission electron microscopy and tomography**
For each mouse strain and acquisition scheme, data was collected on samples derived from at least three different mice.

**Methods: Analysis of connector segmentation**
Of the 18 Volta phase plate tomograms of wild type ROS acquired, five tomograms were selected for analysis for the connector segmentation based on good IMOD tilt-series alignment scores and visual confirmation of well-resolved densities between ROS disks.

**Statistical reporting**

- Statistical analysis methods should be described and justified
- Raw data should be presented in figures whenever informative to do so (typically when N per group is less than 10)
- For each experiment, you should identify the statistical tests used, exact values of N, definitions of center, methods of multiple test correction, and dispersion and precision measures (e.g., mean, median, SD, SEM, confidence intervals; and, for the major substantive results, a measure of effect size (e.g., Pearson's r, Cohen's d)
- Report exact p-values wherever possible alongside the summary statistics and 95% confidence intervals. These should be reported for all key questions and not only when the p-value is less than 0.05.

Please outline where this information can be found within the submission (e.g., sections or figure legends), or explain why this information doesn't apply to your submission:

**Figure 1 - S5** lists the Mean, the SD and the total number of data points for the measured membrane distances shown in Figure 1D. Furthermore, it indicates in how many different tomograms the distances were measured.

**Figure 2** is the only figure with statistical tests. As described in the methods section Analysis of connector segmentation, 7000 connectors were segmented in five Volta phase plate tomograms. 800 were assigned as disk rim connectors and 6200 as disk interior connectors (Table 1). Figure 2E-G show histograms of connector properties and the arrow heads indicate the Median values. Figure 2H shows the connector density per area of disk membrane which was calculated separately for each of the five tomograms. The bar plot indicates the Mean value with the SD as error bars. All P values were calculated according to the two-sample Kolmogorov-Smirnov test.

**Figure 2 - S2D** left panel lists the number connectors for one membrane pair with three different segmentation methods and the right panel the Mean and SD of the respective connectors.

(For large datasets, or papers with a very large number of statistical tests, you may upload a single table file with tests, Ns, etc., with reference to sections in the manuscript.)

**Group allocation**

- Indicate how samples were allocated into experimental groups (in the case of clinical studies, please specify allocation to treatment method); if randomization was used, please also state if restricted randomization was applied
- Indicate if masking was used during group allocation, data collection and/or data analysis

Please outline where this information can be found within the submission (e.g., sections or figure legends), or explain why this information doesn't apply to your submission:

No experimental groups were used in this study.

**Additional data files ("source data")**

- We encourage you to upload relevant additional data files, such as numerical data that are represented as a graph in a figure, or as a summary table
- Where provided, these should be in the most useful format, and they can be uploaded as "Source data" files linked to a main figure or table
- Include model definition files including the full list of parameters used
- Include code used for data analysis (e.g., R, MatLab)
- Avoid stating that data files are "available upon request"

Please indicate the figures or tables for which source data files have been provided:

| |
|---|
| Two representative 4x binned tomograms for each mouse strain or acquisition scheme were deposited in the Electron Microscopy Public Image Archive (EMPIAR). The accession codes are specified in Table 1. |

eLife Sciences Publications, Ltd is a limited liability non-profit non-stock corporation incorporated in the State of Delaware, USA, with company number 5030732, and is registered in the UK with company number FC030576 and branch number BR015634 at the address 1st Floor, 24 Hills Road, Cambridge CB2 1JP | August 2014
